# Hydrodynamic Efficiency and Wake Interactions in Fish School Swimming

**DOI:** 10.3390/biomimetics11040278

**Published:** 2026-04-17

**Authors:** Haoran Huang, Zhenming Yang, Junkai Liu, Jianhua Pang, Zongduo Wu, Hangyu Wen, Shunjun Li

**Affiliations:** 1 School of Mechanical Engineering, Guangdong Ocean University, No. 1 Haida Road, Mazhang District, Zhanjiang 524088, Chinayeungjammy@163.com (Z.Y.);; 2 Shenzhen Institute of Guangdong Ocean University, No. 3 Binhai 2nd Road, Dapeng New District, Shenzhen 518120, China; 3 Guangdong Provincial Key Laboratory of Intelligent Equipment for South China Sea Marine Ranching, Guangdong Ocean University, No. 5, Haibin Avenue Middle, Development District, Zhanjiang 524088, China

**Keywords:** immersed boundary-lattice Boltzmann method (IB-LBM), school swimming, hydrodynamic performance, wake interactions, channel effects

## Abstract

The mechanism by which fish enhance hydrodynamic performance through collective swimming is a research hotspot in the field of underwater bionic robots. This study employs the Immersed Boundary-Lattice Boltzmann Method (IB-LBM) to conduct numerical simulations on a two-dimensional, single-degree-of-freedom (1-DOF) autonomous propulsion bionic fish swarm. It systematically investigates the effects of swarm size and inter-individual spacing on swimming speed and cost of transport (CoT) under two typical configurations: series and parallel arrangements. Findings reveal that hydrodynamic benefits are highly dependent on the spatiotemporal evolution of flow field structures. In the series configuration, an optimal spacing range of 1.5 L to 2.0 L exists within the school, where the “wake capture” effect is pronounced. Trailing fish achieve a maximum speed increase of approximately 41.1% while significantly reducing energy consumption. However, as spacing increases to 2.5 L, the cooperative gain for front and middle-row individuals rapidly diminishes, and the lead fish even experiences significant performance loss. Uniquely, the trailing fish in the four-fish formation exhibits distinct flow field reorganization and performance recovery at the 4.5 L trailing position. In the parallel formation, the “channel effect” and “blocking effect” of the fluid dominate. The study identifies 0.4 L laterally as the critical instability spacing under the investigated kinematic regime, where strong destructive interference causes a sharp deterioration in individual swimming performance. Additionally, the parallel formation exhibits pronounced positional differentiation. Central individuals, constrained by dual lateral flow fields, experience restricted lateral wake expansion and accelerated energy dissipation, resulting in significantly weaker escape capabilities from low-speed conditions compared to marginal individuals. The vortex-dynamic mechanism revealed herein provides theoretical foundations for formation control in multi-fish biomimetic cooperative systems.

## 1. Introduction

Fish schooling behavior offers numerous advantages, including enhanced defense against predators, increased foraging efficiency, and improved endurance [1]. Researchers focus on studying collective motion primarily due to its energy-saving benefits. The advantages of schooling behavior can be attributed to vortex effects and slipstream effects. Mutual vortex interactions within a group improve individual performance, enabling sustained high swimming speeds and enhanced locomotive efficiency through organized vortex structures [2,3,4]. The slipstream effect arises because individuals within a school experience reduced relative velocity due to increased flow between lateral neighbors [5]. Multiple prior studies, through observation or experimentation, have demonstrated the unique hydrodynamic advantages of fish swimming [6,7]. However, assessing the benefits for individual fish within a school is complex, potentially depending on their spatial position within the group, hierarchical role, and physiological state [8]. The varying benefits for each fish within a school due to differences in spatial configuration involve intricate hydrodynamics [9]. When fish swim in groups, they spontaneously form various formation configurations, including linear, parallel, triangular, diamond, and square arrangements [10]. Deng and Shao employed computational fluid dynamics (CFD) methods to investigate hydrodynamic interactions and fish propulsion mechanisms in diamond formations. By varying lateral and longitudinal spacing between individuals, observing the forces and energy expenditure of rear-row fish. Their findings revealed that fish positioned midway behind the leading fish can efficiently utilize the leading fish’s counter-Karman vortex street. When spacing is too small, downstream fish actually expend more energy; however, at optimal spacing, downstream fish exhibit enhanced propulsion efficiency while significantly reducing power consumption [11]. Dong and Lu investigated fluid-hydrodynamic interactions between two traveling-wave oscillating wing profiles simulating side-by-side swimming fish. They analyzed flow characteristics under different phases and lateral spacing configurations. Results indicated lateral interference reduces swimming power consumption under in-phase conditions while enhancing propulsive force under out-of-phase conditions [12,13]. Channel effects are typically inextricably linked to inter-individual distances. Li et al. demonstrated that different fish schools exhibit varying sensitivities to spacing due to channel effects. For series configurations, minimal spacing between fish yields optimal efficiency, whereas larger spacing is more suitable and efficient for rectangular formations [14]. Chang and Qiao et al. employed the constrained immersion boundary method for two-dimensional simulations to reveal swimming behaviors in rectangular and diamond formations under varying phases and spacings [15]. Recent studies have also investigated how flow velocity variations, obstacles, and complex topography disrupt fish migration [16]. Wake matching enables trailing fish to harvest vortex energy from the leading fish’s Karman vortex street for propulsion. By adjusting phase and spacing variables, researchers explored their relationship with energy savings [17]. Only with appropriate vortex phase matching and spacing can vortex integrity be maintained [18]. Experiments demonstrate that two or more objects in a fish formation typically exhibit reduced cost of transport (CoT), increased thrust, enhanced propulsion efficiency, and improved swimming speed [19,20,21]. The intricate fluid–structure interaction mechanisms of biological propulsion have continually inspired a wide array of innovative underwater bionic vehicles, ranging from highly maneuverable robotic fish to advanced soft robotic jellyfish [22]. Researchers have constrained fish swimming degrees of freedom and analyzed the hydrodynamic mechanisms of flexible bodies through the motion of flexible bodies following cylinders [23,24].

Despite extensive computational fluid dynamics simulations of self-propelled fish schools in recent years, systematic quantitative analyses of critical coupling effects across different group sizes remain scarce. Explorations into the critical instability spacing caused by channel effects in parallel formations and the tail flow reorganization mechanisms in long serial formations remain unexplored. Against this backdrop, this study constructs a two-dimensional, single-degree-of-freedom self-propelled fish school. The study focuses on two typical formations—serial and parallel—systematically scanning flow field evolution across scales from two to four fish. It emphasizes investigating the critical spacing effect transitioning from strong near-field coupling to weak far-field interactions. Through quantitative analysis of instantaneous vorticity fields, pressure contours, and transport cost metrics, this study aims to reveal the nonlinear regulatory mechanisms of group size and spatial configuration on individual hydrodynamic performance, providing theoretical foundations for swarm control strategies in underwater bionic robots.

## 2. Method

### 2.1. Fluid Systems—The Lattice Boltzmann Method

The immersed boundary-lattice Boltzmann method (IB-LBM) is widely used to simulate fluid–structure interaction systems [25]. LBM offers unique advantages in handling compressible fluids at low Mach numbers. Furthermore, because it employs mesoscopic particles to discretize the fluid, it can intuitively handle complex problems such as moving boundary dynamics; these advantages have made LBM a preferred choice for many researchers [26]. In this paper, the multiple-relaxation-time (MRT-LBM) is adopted to describe the fluid motion process, and its equation is as follows [27]:

(1) $$f_{\alpha }\left(r+e_{\alpha }\delta_{t},t+\delta_{t}\right)-f_{\alpha }\left(r,t\right)=-\left(M^{-1}SM\right)\left[f_{\alpha }-f_{\alpha }^{eq}\right],\alpha =0,1,\ldots ,8$$

where  f is the distribution function, r  is the spatial coordinate, $e_{\alpha \;}$ is the discrete velocity, t  represents the time step, M is the  9×9  transformation matrix, S is the relaxation matrix, and $f_{\alpha }^{eq}\;$ is the equilibrium distribution function, whose equation is as follows:

(2) $$f_{\alpha }^{eq}=\rho w_{\alpha }\left[1+\frac{e_{\alpha }\cdot u}{c_{s}^{2}}+\frac{{{(e}_{\alpha }\cdot u)}^{2}}{2c_{s}^{4}}-\frac{u^{2}}{2c_{s}^{2}}\right]$$

where $w_{\alpha }$ is the weight coefficient, ρ  is the fluid density, u is the fluid velocity, $c_{s}\;$ is the lattice sound velocity, and $c_{s}=∆x/(∆t\sqrt{3})$.

(3) $$w_{\alpha }=\{\begin{array}{c} \frac{4}{9}\;\alpha =0\\ \frac{1}{9}\;\alpha =1,\;2,\;3,\;4\\ \frac{1}{36}\;\alpha =5,\;6,\;7,\;8 \end{array}$$

**Figure 1:**
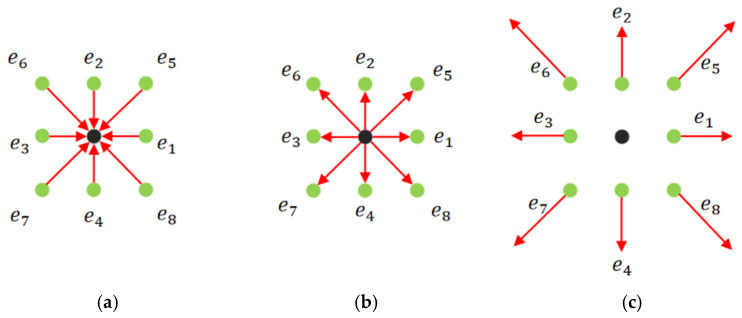
The D2Q9 model before and after collision-streaming: (**a**) before collision; (**b**) after collision; (**c**) after streaming.

In this paper, the D2Q9 model is used to study the two-dimensional fluid solver. Figure 1 shows the D2Q9 model before and after collision-streaming, where the discrete lattice velocity $e_{\alpha }$ is defined as:

(4) $$e_{\alpha }=\{\begin{array}{c} \left(0,0\right)\;\alpha =0\\ (\cos[(\alpha -1)\frac{\pi }{2}],\;\sin\left[\left(\alpha -1\right)\frac{\pi }{2}\right])\;\alpha =1,\;2,\;3,\;4\\ \sqrt{2}(\cos[(2\alpha -1)\frac{\pi }{2}],\;\sin\left[\left(2\alpha -1\right)\frac{\pi }{2}\right])\;\alpha =5,\;6,\;7,\;8 \end{array}$$

The macroscopic physical quantities of fluid density and velocity can be obtained from the distribution function of the fluid particles, and the calculation formulas are as follows:

(5) $$\rho =\sum_{\alpha }f_{\alpha }$$

(6) $$u=\frac{1}{\rho }\sum_{\alpha }f_{\alpha }e_{\alpha }$$

The fluid pressure is related to the fluid density and the lattice sound velocity:

(7) $$p=\rho c_{s}^{2}$$

### 2.2. Fish Body–Fluid Interaction Method—Non-Iterative IBM

In this study, we adopt the non-iterative immersed boundary method (IBM) to handle the fluid–structure interaction at the fish body boundary. This method does not require adding a body force term to the LBM governing equation; instead, it directly modifies the distribution function near the boundary to enforce the no-slip boundary condition. First, the possible distribution function at the Lagrangian points on the fictitious solid boundary is calculated:

(8) $$f_{\alpha }^{*}\left(X\right)=\sum_{x}f_{\alpha }\left(x\right)\delta \left(x-X\right){\left(h\right)}^{2}$$

**Figure 2:**
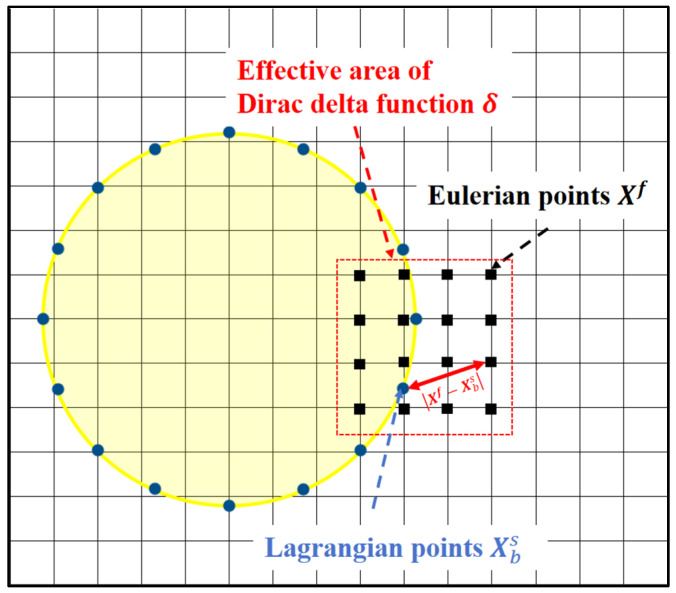
Schematic of the Immersion Boundary Method. Square dots represent Lagrangian grid points, blue circles denote Euler nodes, and the dashed-line region indicates the scope of the Dirac delta function.

In the above equation, as shown in Figure 2, $\delta \left(r_{x},r_{y}\right)\;$ represents the Dirac interpolation function, and  h  denotes the grid spacing, with its expression given by [28]:

(9) $$\delta \left(r_{x},r_{y}\right)=\frac{1}{h^{2}}\xi \left(\frac{r_{x}}{h}\right)\xi \left(\frac{r_{y}}{h}\right)$$

(10) $$\xi \left(r\right)=\{\begin{array}{c} \frac{1}{8}\left(3-2\left|r\right|+\sqrt{1+4\left|r\right|-4r^{2}}\right)\;0\leq \left|r\right|<1\\ \frac{1}{8}\left(5-2\left|r\right|-\sqrt{-7+12\left|r\right|-4r^{2}}\right)\;1\leq \left|r\right|<2\\ 0\;\left|r\right|\geq 2 \end{array}$$

To ensure that the distribution function f within the immersed boundary range satisfies the no-slip boundary condition, the Bounce-Back rule is applied at the solid boundary:

(11) $$f_{\alpha }\left(X\right)=f_{\alpha }^{*}\left(X\right)+2w_{\alpha }\rho \frac{e_{\alpha }\cdot u_{b}}{c_{s}^{2}}$$

$f_{\alpha }\left(X\right)\;$ represents the expected distribution function at the discrete boundary point, where $u_{b}\;$ is the velocity of the discrete boundary point. When the distribution function on the solid boundary points acts on the flow field, to eliminate the deviation between the solid–fluid system, a parameter  λ  is introduced to adjust the LBM governing equation [29]:

(12) $$\lambda \left(n\right)=\frac{1}{2\rho d_{s}({h)}^{2}\sum_{x}\sum_{X}\delta \left(x-X\right)\delta \left(x-X\right)}$$

(13) $${∆f}_{\alpha }\left(x\right)=\sum_{X}\lambda \left(n\right)\left(f_{\alpha }\left(X\right)-f_{\alpha }^{*}\left(X\right)\right)\delta \left(x-X\right)d_{s}$$

where $\;d_{s}\;$ represents the arc length between two adjacent discrete boundary points. The corrected distribution function is distributed to the flow field; thus, the new distribution function in the flow field is expressed as ${\;f}_{\alpha }\left(X\right)+{∆f}_{\alpha }\left(x\right)$. The new distribution function aims to update the macroscopic flow field information, such as macroscopic fluid density and velocity.

### 2.3. Numerical Methods for Fish Body Deformation

**Figure 3:**
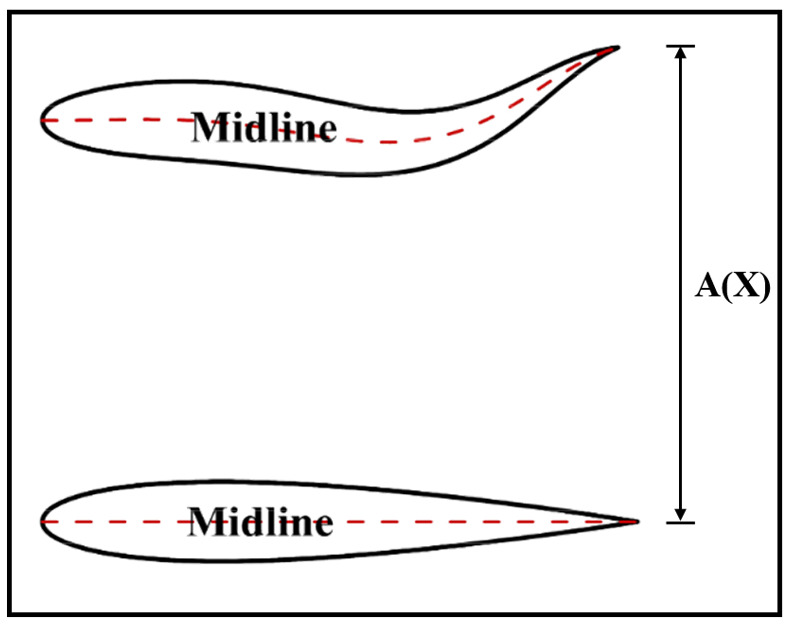
Schematic diagram of midline deformation in fish.

Fish body deformation motion is typically achieved through deformation along the fish’s midline [30], as shown in Figure 3. The general midline displacement equation describing fish body motion is as follows:

(14) $$h_{l}\left(x_{l},t\right)=A\left(x\right){\left(\frac{x_{l}}{L}\right)}^{2}\sin\left(2\pi \left(\frac{x_{l}}{\lambda }-\frac{t}{T}\right)+\varphi \right)$$

(15) $$A\left(x\right)=a_{0}+a_{1}\left(\frac{x_{l}}{L}\right)+a_{2}{\left(\frac{x_{l}}{L}\right)}^{2}$$

where ${\;x}_{l}\;$ represents the horizontal coordinate of the fish body centerline, L  is the arc length of the centerline, t  is the time step, A(x)  denotes the amplitude of the body undulation, λ  is the body wave wavelength, T is the undulation period, φ  represents the initial phase of the oscillation, and $a_{0}$, $a_{1}$, $a_{2}$ are constant coefficients. In this work, the NACA0012 airfoil is adopted as a simplified fish body model, where the airfoil profile represents the curved surface shape of the fish. The half-thickness of the fish body, $d_{l\;}$ can be approximated by a fourth-order polynomial:

(16) $$\frac{d_{l}}{L}=0.2610\sqrt{\frac{x_{l}}{L}}-0.3112\left(\frac{x_{l}}{L}\right)+0.1371{\left(\frac{x_{l}}{L}\right)}^{2}-0.0791{\left(\frac{x_{l}}{L}\right)}^{3}-0.0078{\left(\frac{x_{l}}{L}\right)}^{4}$$

where $\;d_{l\;}$ represents the half-thickness of the fish body at the centerline coordinate. The overall motion of the fish body is governed by Newton’s second law of motion, expressed mathematically as:

(17) $$F_{s}=m\frac{d^{2}r_{c}}{dt^{2}}$$

where $\;F_{s}\;$ represents the fluid force acting on the fish body, and $\;r_{c}\;$ is the translation of the fish body’s center of mass. To avoid collisions and separation among individuals in the fish school during swimming, this paper restricts the motion in the deflection direction. A two-dimensional, one-degree-of-freedom (1-DOF) self-propelled fish model is adopted, which allows the fish to move only in the streamwise direction (transverse direction in global coordinates is constrained). Consequently, the above equation is simplified to:

(18) $$F_{sx}=m\frac{d^{2}r_{cx}}{dt^{2}}$$

To balance the controllability of computational model complexity with the convenience of subsequent quantitative analysis, the primary variables in this study are school size and inter-individual spacing within the school. Spacing varies from 0.3 L to 1.0 L in increments of 0.1 L. This study employs a series of metrics to quantitatively characterize fish swimming performance, primarily including the mean cruising speed $\bar{U}$ and the cost of transport CoT. The swimming speed of a self-propelled fish is dynamically determined by the balance between the hydrodynamic thrust and drag. The instantaneous net force in the swimming direction determines the acceleration of the individual: $m\frac{dU}{dt}=F_{thrust}(t)-F_{drag}(t)$. In a fish school, hydrodynamic interactions alter the localized pressure and velocity fields, resulting in differences in the total thrust and drag experienced by each fish compared to a solitary state. This variation in net force directly establishes a new equilibrium mean swimming speed, calculated over a stable oscillation period T [31]:

(19) $$\bar{U}=\frac{1}{T}\int_{t}^{t+T}U\left(t\right)dt$$

The energy expenditure is evaluated through the input power P(t), which represents the rate of work done by the fish deforming against the surrounding fluid. It is computed by integrating the dot product of the local fluid force $F_{s}$ and the surface deformation velocity $V_{b}$ over the entire fish body:

(20) $$P(t)=-\int_{S}F_{s}\cdot V_{b}ds$$

The cost of transport CoT characterizes the energy consumed per unit distance traveled. Using the time-averaged input power $\bar{P}=\frac{1}{T}\int_{t}^{t+T}P\left(t\right)dt$, and considering that all individuals within the group possess identical body sizes and mass m, the dimensionless CoT formulation is extended and simplified as follows [32]:

(21) $$CoT=\frac{\bar{P}}{m\bar{U}}\approx \frac{\bar{P}}{0.5\rho L^{3}/T^{2}\bar{U}}$$

Calculations show the single-fish average cruising propulsion speed $U/U_{0}=0.7580$, transport cost ${CoT}_{s}=0.4211$. Furthermore, to systematically quantify the energy conversion mechanism and evaluate the hydrodynamic efficiency of the deforming fish body, this paper adopts the energy quantification framework prevalent in the study of oscillating propulsors and deformable airfoils [33]. The performance is dynamically characterized by the time-averaged thrust coefficient ${\bar{C}}_{T}$ and the input power coefficient ${\bar{C}}_{P}$, which are defined as follows:

(22) $${\bar{C}}_{T}=\frac{{\bar{F}}_{thrust}}{0.5\rho U_{0}^{2}L}$$

(23) $${\bar{C}}_{P}=\frac{\bar{P}}{0.5\rho U_{0}^{3}L}$$

Here, $U_{0}$ represents the reference velocity of the fluid. Consequently, the Froude propulsive efficiency η, a critical parameter indicating the ratio of useful hydrodynamic work performed by the fish to the total internal energy expended through structural deformation, is formulated as:

(24) $$\eta =\frac{{\bar{C}}_{T}\bar{U}/U_{0}}{{\bar{C}}_{P}}=\frac{{\bar{F}}_{thrust}\bar{U}}{\bar{P}}$$

These metrics systematically complement the CoT, providing a complete energetic profile of the collective swimming configurations.

### 2.4. Numerical Validation and Independence Studies

To ensure the accuracy and reliability of the numerical results presented in this study, the in-house C++ fluid–structure interaction solver based on the IB-LBM framework underwent rigorous validation prior to conducting the fish school simulations. The verification process consisted of two canonical benchmark cases designed to independently assess different computational modules.

First, the classical problem of flow past a stationary circular cylinder was simulated to verify the fundamental accuracy of the D2Q9 fluid solver and the static implementation of the non-iterative immersed boundary method. In this case, different Reynolds numbers are designed to pass through a fixed cylinder. The Reynolds number was defined as $Re=\rho U_{0}D/\mu$, where ρ is the characteristic fluid density, $U_{0}$ is the far-field fluid velocity, D is the cylinder diameter, and μ is the fluid viscosity. The cylinder diameter was defined as D=20 for grid spacing, and the computing domain size was 40 D×20 D. The average drag coefficient $C_{D}$ and the lift coefficient $∆C_{L}$ between peaks were calculated for comparison. As shown in Table 1, the mean value of the drag coefficient and the amplitude between the peaks of the lift coefficient are all highly consistent with the data in the literature. The drag coefficient $C_{D}$ and the dimensionless lift coefficient $C_{L}$ in the dimensionless form are shown in Equation (25), and the Strouhal number $S_{t}$ is defined as follows:

(25) $$C_{D}=\frac{F_{D}}{0.5\rho U_{0}^{2}D},\;C_{L}=\frac{F_{L}}{0.5\rho U_{0}^{2}D},\;S_{t}=\frac{fD}{U_{0}}$$

**Figure 4:**
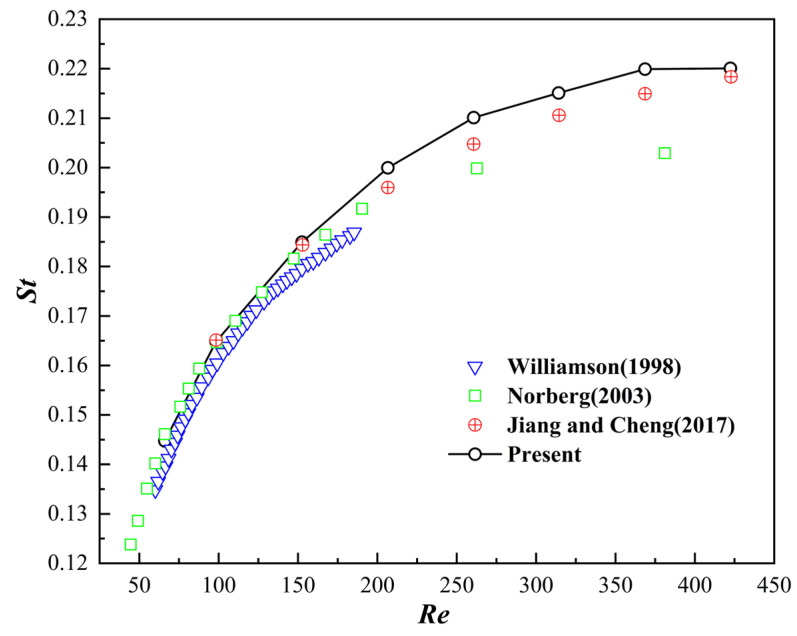
Flow around a circular cylinder at different Reynolds numbers and the relationship with the Strouhal number [37,38,39].

As shown in Figure 4, the calculated drag coefficients and Strouhal numbers across a range of low to moderate Reynolds numbers exhibited excellent agreement with established experimental and numerical data in the literature.

Second, the dynamic moving boundary processing and unsteady force quantification were validated through the simulation of an actively flapping airfoil. In this paper, the NACA0012 airfoil is selected as the validation object and placed in a uniform flow to perform flapping motion. The kinematics of this airfoil are described by both heave displacement and pitch angle variation, with the specific expressions as follows:

(26) $$\theta (t)=\theta_{0}cos(2\pi ft)$$

(27) $$h(t)=h_{0}cos(2\pi ft+\phi )$$

In the formula, $\theta_{0}$ and $h_{0}$ represent the maximum pitch angle and maximum heave displacement, respectively, while f and ϕ denote the flapping frequency and phase angle. The computational conditions are set as follows: the airfoil is placed in a uniform flow field with a Reynolds number Re = 1000, and the pivot point of the airfoil is located at 0.3 L from the leading edge, where L is the chord length of the airfoil. The maximum pitch angle $\theta_{0}=10{}^{\circ}$, the maximum heave amplitude $h_{0}=0.5\;L$, the phase lag ϕ=90°, and the dimensionless frequency $f^{*}=fL/U_{0}$, where $U_{0}$ is the freestream velocity. The computational domain size is 20 L×20 L, and the grid size is L=100/Δx.

In this paper, the lift coefficient, drag coefficient, moment coefficient, and hydrodynamic power coefficient of the propulsive wing are calculated. The specific definitions of the moment coefficient and hydrodynamic power coefficient are given by the following formulas:

(28) $$C_{M}=\frac{M_{Z}}{0.5\rho U_{0}^{2}L^{2}}$$

(29) $$C_{P}=\frac{P}{0.5\rho U_{0}^{3}L}$$

**Figure 5:**
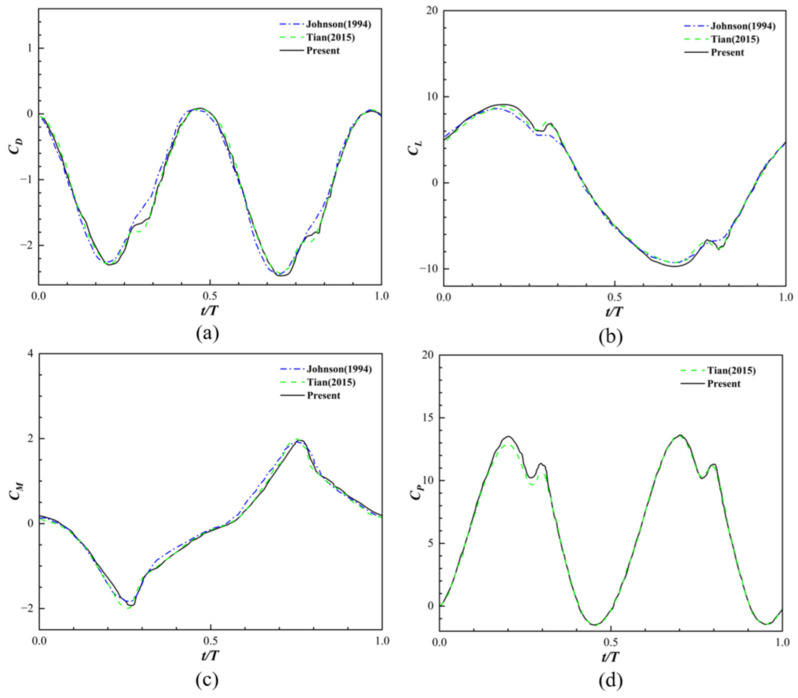
(**a**) drag coefficient; (**b**) lift coefficient; (**c**) moment coefficient; (**d**) hydrodynamic power coefficient [40,41].

As shown in Figure 5, due to the symmetry of the flapping mode, both the lift coefficient and the moment coefficient exhibit symmetric distributions about the zero axis. Further analysis of the frequency characteristics reveals that the fluctuation frequency of the drag coefficient is twice that of the lift coefficient, the frequency of the hydrodynamic power coefficient is twice that of the moment coefficient, and the drag coefficient and the hydrodynamic power coefficient share the same frequency. The time-varying thrust and power coefficients accurately captured the generation and shedding of the counter-Karman vortex street, closely matching the reference benchmark results [40,41].

**Figure 6:**
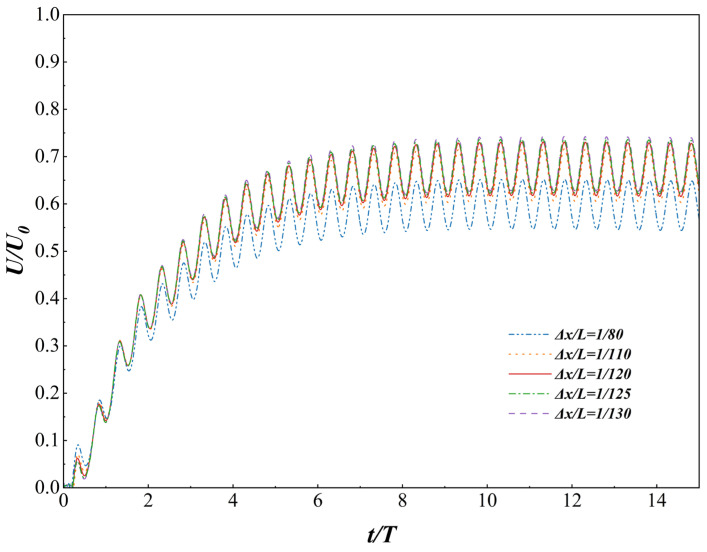
Dimensionless transient velocity ($U/U_{0}$) versus dimensionless time (t/T) under five different spatial resolutions.

Finally, to ensure the accuracy and reliability of the numerical results, a systematic study of grid independence was conducted using a single autonomous swimming fish model prior to the multi-fish simulations, in order to eliminate any potential numerical errors. This paper evaluates five spatial grid densities defined by the feature length L: Δx/L=1/80, 1/110, 1/120, 1/125, and 1/130. As shown in Figure 6 below, this figure illustrates the forward speed of the fish movement model at different grid resolutions. The Reynolds number was set as Re = 300. The motion parameters were A = 0.11 L, λ=1.0 L and T = 300 ∆t. The results showed that the convergence state was reached when the grid resolution was ∆x/L = 1/120. While the coarse mesh (Δx/L=1/80) exhibits a slight deviation, the macroscopic performance metrics plateau for resolutions finer than 1/110. The deviation in the mean cruising speed between Δx/L=1/120 and the finest grid 1/130 is remarkably negligible (under 1.0%). Consequently, the grid resolution of Δx/ L=1/120 was adopted for all subsequent multi-fish simulations to balance computational fidelity with efficiency.

These systematic validations confirm that the numerical platform possesses sufficient fidelity and robustness to accurately resolve the complex vortex dynamics and hydrodynamic interactions inherent in multi-fish schooling scenarios.

## 3. Results and Discussion

### 3.1. Performance of a Single Fish During Steady Swimming

**Figure 7:**
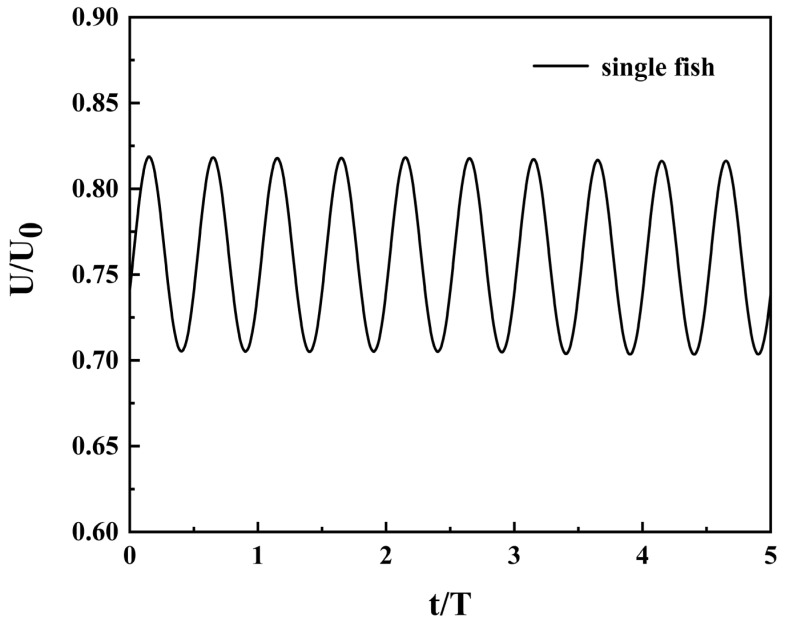
Transient velocity during autonomous swimming of a single fish.

**Figure 8:**
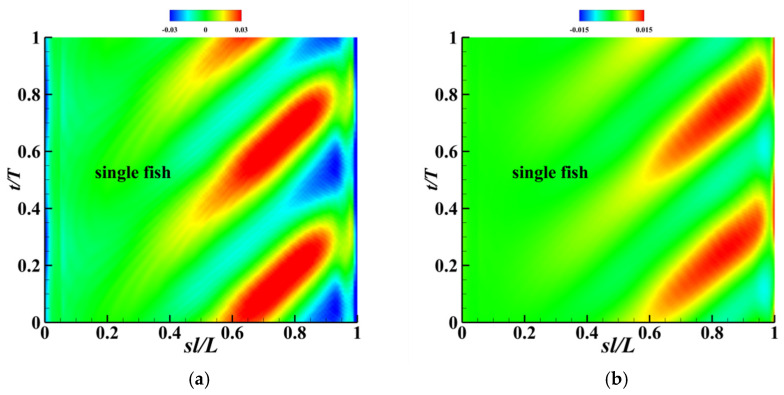
(**a**) Spatiotemporal distribution of thrust along the x-direction for each segment of the fish body; (**b**) Spatiotemporal distribution of power consumption for each part of the fish body.

This section first conducts numerical simulations of autonomous swimming by a single fish to obtain its fundamental hydrodynamic characteristics, providing a critical comparative benchmark for subsequent studies on the fluid effects of cooperative swimming by multiple fish. Figure 7 displays the instantaneous velocity variation curve during the autonomous swimming of a single fish. After an acceleration phase of approximately 30 cycles, the fish body reaches a steady-state cruising phase. At this point, the instantaneous velocity exhibits periodic sinusoidal oscillations synchronized with the fish body’s oscillation frequency. As fluid flows over the fish body surface, a pressure difference is generated, inducing a lateral force on the fish. Figure 8a depicts the spatiotemporal distribution of thrust along the x-direction, where the red regions quantify the instantaneous thrust generated by each segment, and the blue regions represent the corresponding instantaneous drag experienced by each segment. This distribution clearly indicates that the primary contribution to propulsive force is concentrated in the rear section of the fish body, particularly the tail fin region extending from 0.5 L to 0.95 L. The two largest thrust peaks occur near 0.75 L. The large amplitude of the tail swing efficiently promotes the formation of counter-rotating Karman vortices, generating net thrust to overcome the drag dominated by the anterior body. Figure 8b shows the spatiotemporal distribution of power consumption in the fish body. Power dissipation exhibits a traveling wave pattern similar to force generation, with high-power consumption regions being longer and narrower than high-thrust regions. Red regions indicate localized power consumption (positive power) from active muscle contraction, while blue regions represent passive energy recovery (negative power) from the surrounding fluid. This spatiotemporal non-uniformity of positive and negative power consumption reflects the fundamental characteristic of efficient propulsion achieved through energy exchange mechanisms between the fish’s elastic structure and the fluid. The tail region serves as both a high-thrust generation zone and the primary energy dissipation area, while the blue sections in the body’s tail exhibit a certain capacity for energy recovery.

### 3.2. Hydrodynamic Performance of Individuals in Series Fish Schools

**Figure 9:**
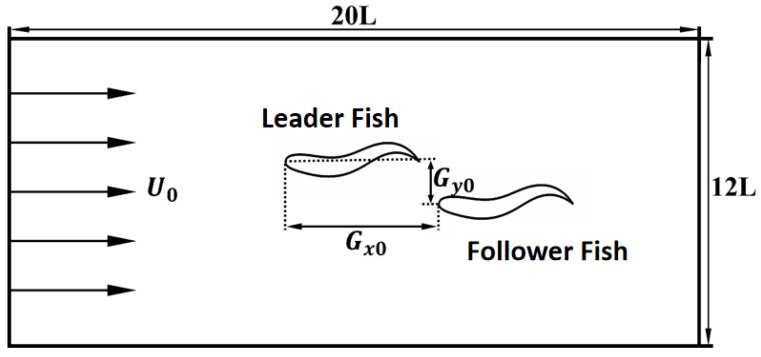
Schematic diagram of the fish school computational domain.

In this subsection, we discuss the simulation results of the fish school, examining whether changes in the school structure produce cumulative effects by analyzing both the fish school subsystem and the school itself. First, we place the fish school within a computational domain of 20 L × 12 L, as shown in Figure 9. To ensure numerical stability and physical accuracy, specific boundary conditions are strictly enforced: a uniform velocity boundary condition ($u=U_{0},v=0$) is prescribed at the inlet, and a convective boundary condition (∂u/∂x=0) is implemented at the outlet to allow shedding vortex structures to exit the domain smoothly without non-physical numerical reflection. Furthermore, free-slip boundary conditions are applied to the top and bottom boundaries. The transverse domain width of 12 L ensures a minimal blockage ratio, effectively eliminating artificial flow acceleration caused by lateral wall confinement. Guided by the rigorous grid-independence study, the spatial lattice resolution is firmly set to Δx/L=1/120, and the temporal resolution is defined by the discrete time step *∆*t, with the body wave period set to T=300∆t.

Considering that fish swimming side by side may produce repulsion and attraction effects, the macroscopic motion of the fish bodies is strictly confined to the flow direction (x-direction). The Reynolds number is set to Re = 300, defined as *Re* = *ρU_0_L/μ*. All fish groups share identical kinematic parameters: maximum tail amplitude A=0.11L, body wave period T, and wavelength λ=1.0L, and an identical initial phase (φ=0). Consequently, all individuals within the school oscillate strictly in-phase throughout the simulation. While phase difference is known to be a critical factor for optimal wake energy harvesting, it was deliberately kept constant in this specific study. This methodological choice was made to mathematically decouple the kinematic variables, allowing us to strictly isolate and quantify the fundamental hydrodynamic phenomena—such as the pure channel effect and baseline wake capture—driven solely by spatial configuration (spacing) and group size. Introducing phase delay as an independent variable for multi-fish systems would trigger a combinatorial explosion of parameters, which is beyond the scoped objective of evaluating geometric interference.

To ensure robust data collection and evaluate the stabilized hydrodynamic performance, strict convergence criteria are applied. All simulations are executed for a total of 70 oscillation cycles. Quantitative measurements (such as mean speed and cost of transport) are extracted only after the system reaches a stable quasi-steady state, which is achieved following an initial transient phase of 50 cycles. The final macroscopic metrics are mathematically time-averaged over an exact window of 4 consecutive oscillation cycles. In this case, only the initial spacing between the Follower Fish and Leader Fish is varied. For the serial experiment, $G_{y0}=0$, and the inter-individual spacing parameter $G_{x0}$ is scanned through values of 1.5, 2.0, 2.5, 3.0, 3.5, 4.0, and 4.5. The experimental subjects encompassed three distinct group sizes: two fish, three fish, and four fish.

**Figure 10:**
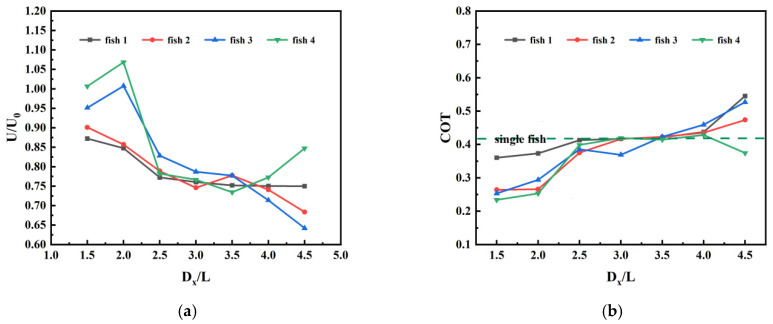
Performance of leader and follower fish at different initial spacings in series modal configuration: (**a**) Swimming speed; (**b**) Transportation cost.

**Figure 11:**
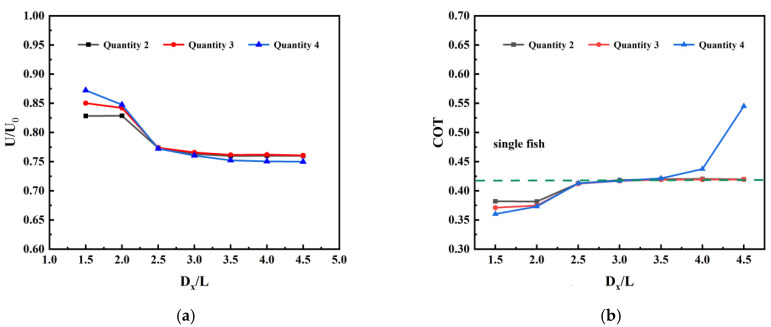
Leader fish performance under different group sizes in series mode: (**a**) swimming speed; (**b**) transport cost.

**Figure 12:**
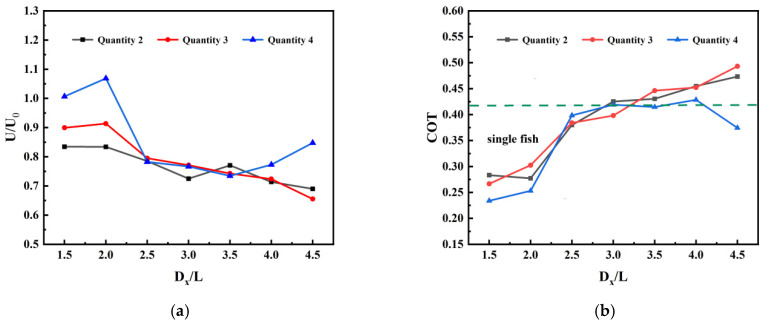
Follower fish performance under different group sizes in series mode: (**a**) swimming speed; (**b**) transport cost.

Due to the strong fluid–structure interaction occurring when fish schools swim in flow fields, the hydrodynamic performance of individual fish is significantly influenced by spatial distance. Different school sizes also generate more complex hydrodynamic effects. This section investigates the coupled effects of two parameters—individual count and spatial separation—under typical tandem swimming configurations. Figure 10, Figure 11 and Figure 12 illustrate the time-averaged hydrodynamic performance of individual fish within the school as functions of both individual count and spatial separation.

Figure 10a illustrates the relationship between individual swimming speed and spatial distance when four fish swim in series. Figure 10b depicts the relationship between individual transport cost and spatial distance during series swimming of four fish. As shown in Figure 10, the fish group exhibits significant non-monotonic differences at different spacing intervals. When the inter-fish spacing is at the smaller intervals of 1.5 L and 2.0 L, the swimming speed of fish in the tandem formation significantly increases while the transport cost decreases, indicating that the group swimming formation generates positive hydrodynamic benefits for all members at these spacing intervals. Notably, trailing fish 3 and fish 4 exhibit substantial speed increases. Fish 4 reaches peak speed at 2.0 L, achieving approximately 41.1% higher velocity than solitary swimming while reducing transport cost by about 45.2% at 1.5 L—significantly lowering energy expenditure. This demonstrates pronounced close-formation effects and exceptionally high efficiency in serial swimming. However, as the spacing increased to 2.5 L, the efficient cooperative mode of the school deteriorated markedly. The speeds of Leader Fish 1, Follower Fish 2, and Follower Fish 3 rapidly declined, accompanied by increased transport costs. This signaled the closure of the “high swimming speed, low energy consumption” golden window, shifting the system from cooperative profit to a state with no significant benefit. When spacing further increased to 3.5 L, the swimming speeds of individuals in the front and middle rows generally fell below the single-fish baseline. At 4.5 L spacing, Leader Fish 1 exhibited a slight decrease of approximately 1.1% in swimming speed compared to single-fish swimming, yet transport costs surged by about 31.0%, with swimming energy consumption skyrocketing. This indicates that at large spacing, front-to-middle-row fish not only lose synergistic gains but also face increased propulsion resistance due to flow field interference, low-efficiency swimming. In stark contrast, the trailing fish 4 exhibited a unique performance reversal: although its speed briefly bottomed out at 3.5 L, it rebounded at 4.5 L, achieving approximately 12.1% higher swimming speed than a single fish while reducing transport costs by about 11.9%. This phenomenon demonstrates that at long intervals, rear individuals can still overcome inefficiency and gain hydrodynamic benefits. These results indicate that the optimal initial spacing range for a four-fish series formation is 1.5 L to 2.0 L, where the drafting effect is most pronounced. In practical long-formation swimming, excessive spacing among front- and middle-row individuals should be avoided.

Figure 11 illustrates how the swimming speed of the leading fish and the transport cost vary with spatial distance when fish schools of different sizes swim in series. Experimental data reveal that the presence of the trailing school exerts a significant hydrodynamic influence on the leading fish ahead, with the efficiency of this effect being jointly regulated by both school size and spatial distance. It can be observed that the trends in swimming speed and transport cost for different numbers of fish swimming in a series formation exhibit high consistency. When the spatial distance between fish bodies is 1.5 L, the swimming speed of the leading fish in a two-fish formation is approximately 0.8382, representing an increase of about 9.33% compared to a single fish, while the transport cost is approximately 0.3820, a reduction of about 9.28% compared to a single fish. When the group size increased to three fish swimming, the lead fish’s speed rose to approximately 0.8502, an increase of about 12.22% compared to a single fish, with transport cost at approximately 0.3710, a reduction of about 11.88%; When the group size increases to four fish swimming, the lead fish’s swimming speed further increases to approximately 0.8722, representing an improvement of about 15.13% compared to a single fish. The transportation cost is approximately 0.36, a reduction of about 14.44%. This phenomenon strongly demonstrates that within the near-field range, the swimming of rear fish effectively improves the wake environment for the leading fish in the front row. Moreover, the greater the number of following fish, the more significant the near-field hydrodynamic gain obtained by the leading fish. However, as the spacing increases, this scale-based gain effect rapidly diminishes. When the spacing exceeds 2.5 L, the positive gain from the rear fish group to the leading fish essentially disappears. The swimming speed of the lead fish gradually declines to the level of a solitary fish. Yet, when the spacing exceeds 4.5 L, the transport cost significantly increases by approximately 29.46%. This negative effect indicates that in long-formation, large-spacing configurations, the complex flow field generated by multiple trailing fish triggers adverse negative feedback mechanisms in the far field. This forces the lead fish to expend substantial additional energy to maintain stable propulsion. In summary, for the lead fish, the “hydrodynamic profit from the rear group” is primarily concentrated within the near field (within 2.0 L), with greater benefits observed at larger group sizes. Conversely, in the far field with large spacing, an excessively large group size evolves into a hydrodynamic burden for the lead fish.

Figure 12 illustrates how the swimming speed and transport cost of the trailing fish at the rear of a series-connected fish school vary with spatial distance across different school sizes. Comparative analysis reveals that the hydrodynamic benefits for individuals at the rear of the formation depend not only on the wake of the fish immediately ahead but are also closely related to the “cumulative flow field history” of the entire upstream school. This exhibits distinct characteristics of “scale gains” and “long-range differentiation”. At a spatial distance of 1.5 L, trailing fish across all sizes exhibit enhanced speeds and substantially reduced transport costs, demonstrating highly efficient propulsion. Larger group sizes yield more pronounced trailing benefits, indicating that in tightly coupled formations, increased numbers enable trailing fish to extract and utilize greater energy advantages from the accumulated wake upstream. However, as spacing progressively increases, the performance of trailing fish across different group sizes gradually converges. At 2.5 L, the swimming speed of trailing individuals in two-, three-, and four-fish systems rapidly declines back toward the single-fish baseline, with transport costs rising accordingly. This further confirms that 2.5 L represents a critical inflection point in flow field evolution. At this point, the ordered vortex structure induced by the upstream fish becomes unstable and damaged, obstructing the wake effect and resulting in the loss of scale advantages. For smaller groups (two-fish and three-fish), the trailing fish exhibit a typical “long-interval decay” pattern. Taking the maximum spacing of 4.5 L as an example, the swimming speeds of the rear fish in both the two-fish and three-fish formations fell below the single-fish baseline, while transport costs increased. This quantitative data indicates that without sufficient upstream disturbance bodies, the wake generated by only 2–3 fish will either completely dissipate or evolve into a highly damped structure after long-distance convection, resulting in severe hydrodynamic losses for the rear fish. In stark contrast, the rear fish in the four-fish formation exhibits a unique “long-range recovery” characteristic. After experiencing a performance trough at a spacing of 3.5 L, its metrics rebound against the trend at spacings of 4.0 L and 4.5 L. This finding holds significant physical implications: it demonstrates that only when the upstream fish group reaches a critical size (e.g., *N* = 4) does the detached wake energy become sufficient to undergo beneficial secondary reorganization and resonance after undergoing far-field convection. This process opens a second efficient swimming window for the individual at the very rear of the formation.

**Figure 13:**
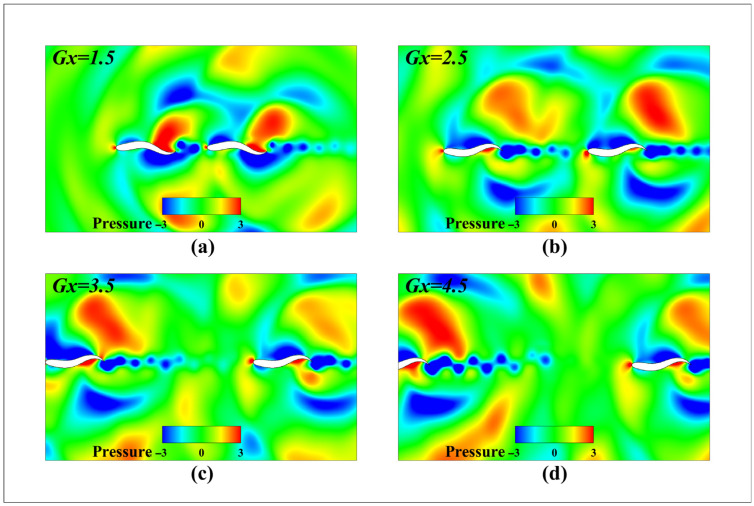
Transient pressure contour map for twin fish with different initial spacing in series modal configuration (**a**) *G_x_* = 1.5 L; (**b**) *G_x_* = 2.5 L; (**c**) *G_x_* = 3.5 L; (**d**) *G_x_* = 4.5 L.

Figure 13 shows the instantaneous pressure contour map for the dual fish system under series mode at different initial spacing, intuitively revealing the mechanism of hydrodynamic interaction as spacing varies. When 1.5 L, the counter-Karman vortex street induced by the leading fish’s tail oscillation exhibits high phase matching with the body-driven flow of the trailing fish, creating a synergistic pressure field effect. The high-pressure fluid pushed backward by the leading fish’s tail fin precisely acts near the suction surface of the trailing fish’s head, generating significant thrust gain. Simultaneously, the low-pressure zone at the trailing fish’s head improves pressure recovery at the leading fish’s tail. As the spacing increases from 2.5 L to 3.5 L, the pressure field coupling efficiency gradually decreases. The high-pressure wake from the leading fish directly impacts the rear fish’s head, creating a large reverse pressure gradient that significantly increases the rear fish’s head-on drag. When the spacing further expands to 4.5 L, the pressure field synergy mechanism completely fails. The leading fish’s tail vortex completely dissipates, with generated energy dissipated in the far field. The trailing fish’s head, exposed to the free-stream flow rather than the low-pressure zone, causes its body’s front-end drag to revert to the single-fish level.

**Figure 14:**
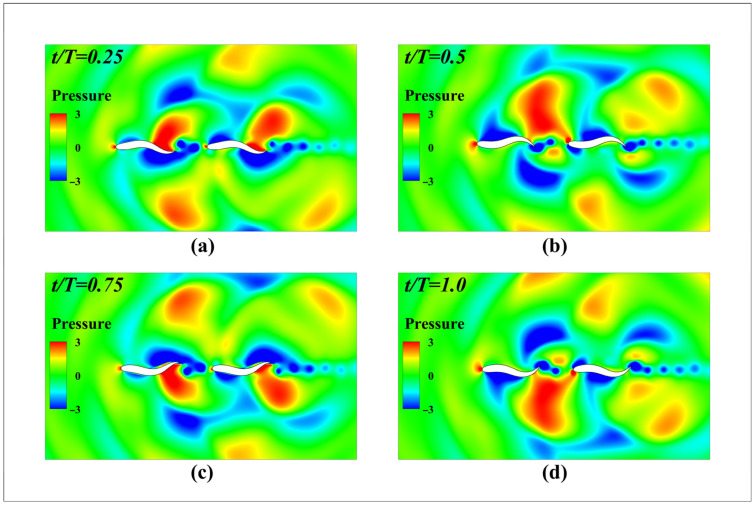
Instantaneous pressure contour map during the double-fish period in series-connected modal analysis: (**a**) *t*/*T* = 0.25; (**b**) *t*/*T* = 0.5; (**c**) *t*/*T* = 0.75; (**d**) *t*/*T* = 1.0.

Figure 14 depicts the instantaneous pressure distribution during the double-fish cycle in series mode. When the leading fish swings its tail upward, the pushing effect on the fluid (or local fluid displacement) creates a high-pressure zone above the tail, while fluid acceleration generates a low-pressure zone below. The trailing fish encounters a reconfigured asymmetric pressure gradient, which, through the Bernoulli effect, converts into a net forward thrust. The reverse Karman vortex street induced by the tail swing further enhances propulsion efficiency. The tail vortex generated by the leading fish creates a periodic velocity gradient field behind it, providing conditions for energy utilization by the trailing fish. This confirms that the wake flow field structure of the leading fish exerts a decisive modulating effect on the instantaneous hydrodynamic performance of the trailing fish.

**Figure 15:**
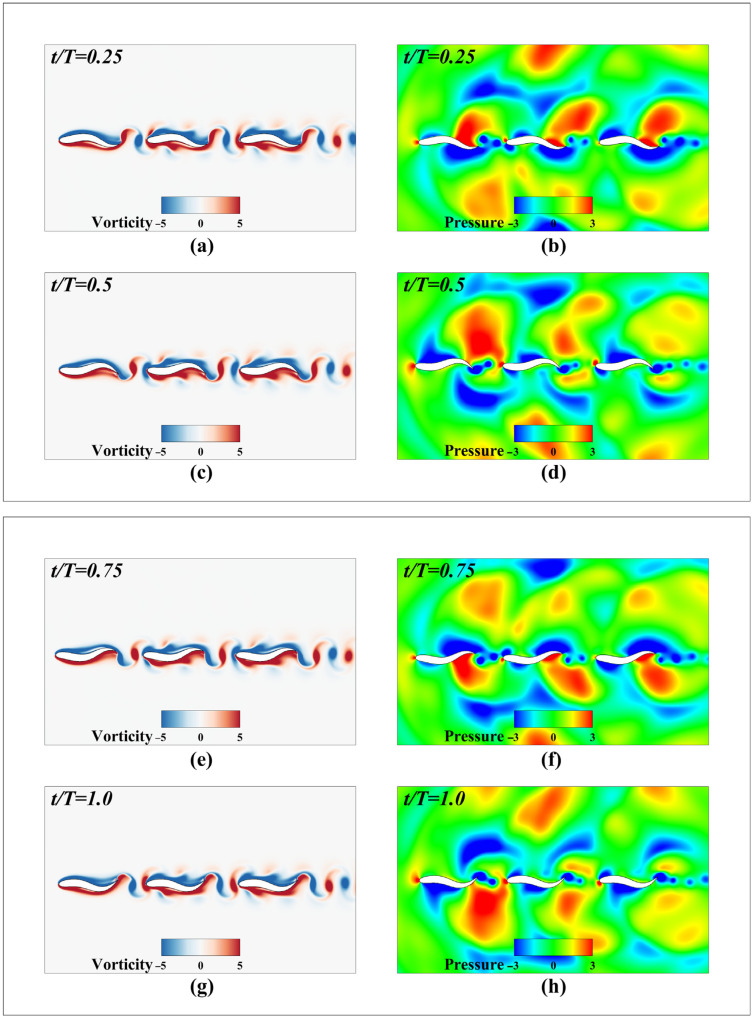
Instantaneous vorticity and pressure cloud maps for three-fish periods in series modal analysis: (**a**) Vortex figure at t/T=0.25; (**b**) Pressure diagram at t/T=0.25; (**c**) Vortex figure at t/T=0.5; (**d**) Pressure diagram at t/T=0.5; (**e**) Vortex figure at t/T=0.75; (**f**) Pressure diagram at t/T=0.75; (**g**) Vortex figure at t/T=1.0; (**h**) Pressure diagram at t/T=1.0.

Figure 15 shows the instantaneous vorticity and pressure fields during the three-fish cycle in series mode. The vorticity map reveals that the vortex shed from the tail of the leading fish (Fish 1) collides with and shears against the leading edge of the middle fish (Fish 2), causing the shed vortex to fragment. This induces the reformation of a secondary complete vortex structure on both the upper and lower surfaces of Fish 2. This intense vortex-fish interaction reconfigures the unique pressure gradient distribution on the fish’s surface. The restructured vortices propagate rearward along the fish’s streamlines, ultimately interacting with the tail vortices. This interaction significantly enhances the counter-Karman vortex street at Fish 2’s tail, increasing thrust. Similarly, the vortex generated at Fish 2’s tail is split by the leading edge of the trailing Fish 3, inducing a similar secondary vortex evolution and wake fusion process on Fish 3’s surface. This further concentrates the energy of the counter-Karman vortex street at Fish 3’s tail, thereby generating higher thrust during propulsion.

It is noteworthy that throughout these visualizations, the instantaneous pressure contours appear more globally distributed and complex compared to the tightly localized vorticity fields. This visual distinction arises from both physical fluid dynamics and the inherent numerical characteristics of the IB-LBM solver. Physically, vorticity is primarily generated at the fluid-solid interfaces due to viscous effects and subsequently convects and diffuses within the immediate wake. In contrast, pressure acts as an elliptic and non-local variable; local kinematic disturbances from the tail oscillations instantaneously alter the pressure gradient across the broader computational domain. Numerically, LBM operates as a weakly compressible solver governed by the state equation $p=\rho c_{s}^{2}$. The dynamic movement and rapid acceleration of the immersed boundaries inevitably generate weak acoustic pressure waves. While these small-scale acoustic fluctuations are a known numerical artifact of IB-LBM that minimally affects the accuracy of the macroscopic incompressible velocity and vorticity fields, they actively superimpose onto the hydrodynamic pressure field, visually contributing to the broader background fluctuations observed in the pressure contours.

**Figure 16:**
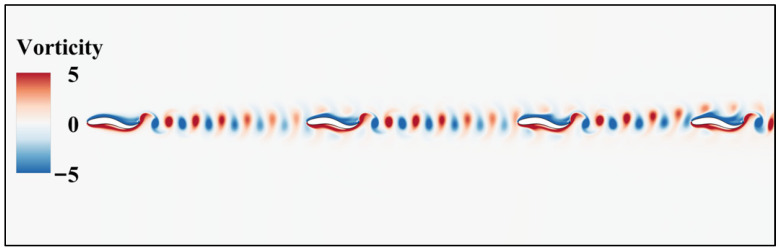
Four-fish instantaneous vorticity cloud map with an initial spacing of 4.5 L in series mode.

**Figure 17:**
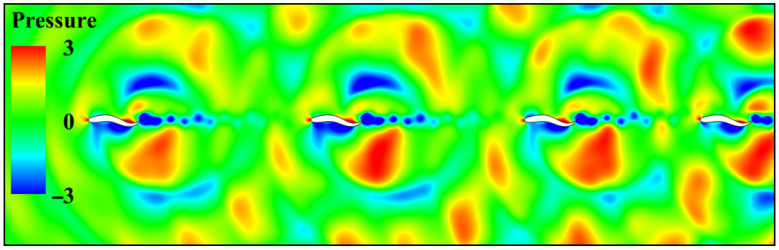
Transient pressure contour map of a four-fish array with an initial spacing of 4.5 L in series mode.

Figure 16 and Figure 17 illustrate the transient flow field characteristics of a four-fish series system at a large spacing of 4.5 L. Although the cooperative gain of the leading fish has largely decayed at this spacing, the fish at the rear of the formation exhibits a unique phenomenon of flow field reconstruction and performance recovery. The vortex shed by the leading fish undergoes significant attenuation after traversing the long convective path at 4.5 L. Trapped within this decaying and turbulent wake, trailing fish 2 and trailing fish 3 generate new vortices through their own oscillations. However, these vortices primarily mix chaotically with upstream fragmented vortex structures, failing to form stable vortex streets. This explains the performance decline of the middle fish at this spacing. However, after continuous disturbance from the first three fish, the flow field undergoes subtle self-repair before reaching the trailing fish (Fish 4). This visually observed “self-repair” mechanism is now quantitatively corroborated by the macroscopic performance metrics previously established in Figure 10. While the intermediate individuals (Fish 2 and Fish 3) suffer severe hydrodynamic degradation due to fragmented upstream vorticity, the reconstituted wake coherence ahead of Fish 4 translates directly into measurable dynamic benefits. The oscillation of the terminal fish matches this reorganized wake, allowing it to extract residual energy from the fluid. This indicates that the complex flow field generated upstream by the fish school successfully reorganized ahead of Fish 4’s head, restoring an efficient anti-Karman vortex shedding pattern. Rather than relying solely on the visual intensity of the contour maps, the dynamic benefits of this flow field reconstruction are strictly quantified by the anomalous performance reversal of Fish 4. Observing the head region of the trailing fish 4, the re-established favorable pressure gradient produces an effect similar to “leading edge suction.” This localized drag reduction and thrust enhancement mathematically culminate in the quantified metrics at the 4.5 L spacing: Fish 4 achieves a 12.1% higher swimming speed than the single-fish baseline, concurrently accompanied by an 11.9% reduction in the cost of transport. These deterministic macroscopic data provide robust quantitative validation that the far-field wake reorganization effectively restores propulsive efficiency at the rear of the long formation.

Around the tail fin of the trailing fish (Fish 4), a well-ordered alternation of high-intensity positive and negative pressure zones forms. This distinct pressure gradient distribution enables more efficient rearward acceleration of the fluid during oscillation, thereby generating greater net thrust. In summary, at the large spacing of 4.5 L, the four-fish system exhibits a flow field maintenance mechanism. Although the wake of a single fish dissipates in the far field, the multi-fish formation achieves a new hydrodynamic gain in the far field through continuous flow field corrections, following an intermediate transitional state. The trailing fish (Fish 4) actually benefits from the cumulative fluid history formed by the preceding three fish. This far-field wake reorganization effect is the fundamental reason why the trailing individual in the four-fish formation outperforms both the intermediate individuals and the trailing individuals in two-fish or three-fish formations.

### 3.3. Hydrodynamic Performance of Individuals in Parallel Fish Schools

**Figure 18:**
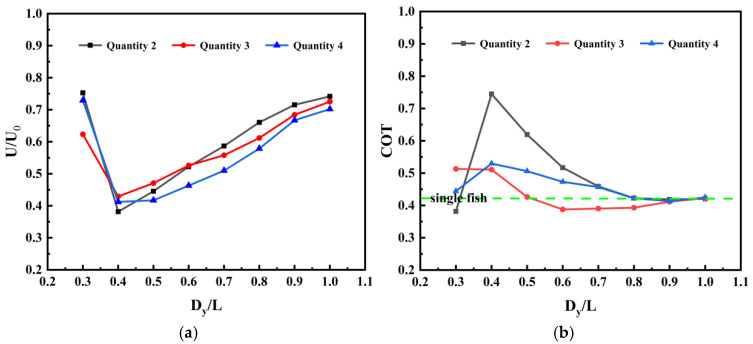
Performance of a fish-following system under parallel mode at different initial spacing: (**a**) swimming speed; (**b**) transportation cost.

Figure 18 illustrates the relationship between the swimming speed of the second-position follower fish and lateral spatial distance for different numbers of fish swimming in parallel formation, as well as the corresponding transport costs. When swimming in parallel formations, schools of varying sizes can maintain a stable geometric configuration, with the entire group moving forward at a consistent average speed. Due to the “channel effect” between parallel formations, significant repulsive and attractive forces arise. In the calculations, fish movement is restricted to the lateral direction only. It can be observed that, unlike the general acceleration phenomenon in series mode, trailing fish in parallel mode exhibit a pronounced speed reduction characteristic under most operating conditions. Furthermore, severe hydrodynamic instability occurs at specific spacing intervals. Comparative analysis reveals that Fish 2’s hydrodynamic performance is highly dependent on the fluid boundary conditions on both sides: its performance as an edge individual is significantly superior to that as an internal sandwich individual. At small spacing ($G_{y0}$ ≈ 0.3 L), positional effects exhibit pronounced differences. In a two-fish formation, Fish 2 experiences interference from only one side of the flow field, demonstrating an exceptional near-wall slip effect. Its swimming speed nearly matches that of a single fish, with slightly reduced transport cost. In the three-fish formation, Fish 2, caught in the “middle squeeze,” suffers severe bilateral fluid blockage. Its speed plummets while transport cost increases. In the four-fish formation, Fish 2 experiences an asymmetric channel effect that alleviates pressure in its flow field, allowing swimming speed to recover but transport cost to rise. However, when spacing increases to the critical point $G_{y0}$ = 0.4 L, Fish 2 in all configurations experiences a “performance collapse.” Swimming speed plummets dramatically regardless of whether neighbors are on one or both sides, indicating that $G_{y0}$ = 0.4 L represents an absolute destructive interference zone for parallel swimming under the current baseline kinematic parameters. It is imperative to note that this specific spacing threshold is not a universal physical constant; variations in phase synchronization, tail amplitude, or Reynolds number would fundamentally alter the shed shear layer dynamics and the transverse wake width, consequently shifting the exact spatial location of this critical instability zone. Energy consumption surges (asymmetric cost). Notably, in three-fish and four-fish arrays, Fish 2 in the middle position swims slower but maintains relatively balanced transport costs. Conversely, Fish 2 in the edge positions of two-fish arrays experiences severe unilateral destructive interference, potentially generating massive deflection torques. This forces it to expend extremely high energy to maintain posture balance, resulting in greater fatigue due to asymmetric forces. When $G_{y0}$ > 0.5 L, the edge effect’s advantage becomes apparent. Fish 2 in the two-fish formation, burdened by lighter “unilateral interference,” exhibits the fastest speed recovery, significantly outperforming the inner Fish 2 in three- and four-fish formations. At $G_{y0}$ = 1.0 L, the edge fish’s speed approaches the single-fish baseline. In contrast, Fish 2 in the three- and four-fish formations remains constrained by “bilateral interference,” exhibiting significantly slower speed recovery compared to the two-fish formation. This indicates that fluid blockage effects within parallel formations exhibit long-range properties, trapping internal individuals within a broad spacing range and preventing escape from the low-speed dilemma caused by the “channel effect”.

**Figure 19:**
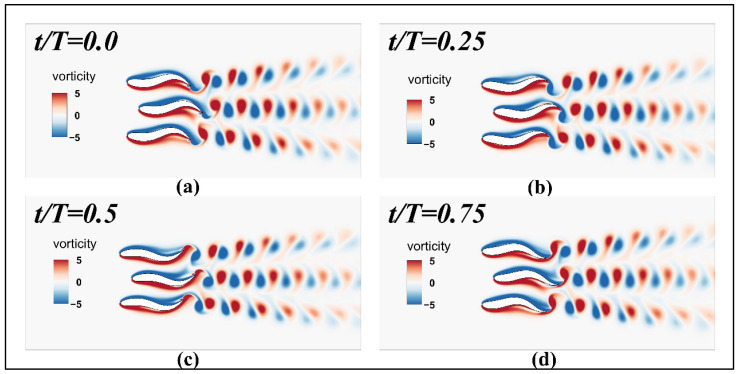
Parallel modal initial spacing 0.4 L periodic instantaneous vorticity contour map for three fish periods: (**a**) t/T=0.0; (**b**) t/T=0.25; (**c**) t/T=0.5; (**d**) t/T=0.75.

Figure 19 shows the instantaneous vorticity contour map for the three fish during the cycle at an initial parallel modal spacing of 0.4 L. The vorticity map at this spacing reveals that the wake structure of the middle fish (Fish 2) exhibits significant spatial confinement due to the excessively narrow spacing, resulting in extremely poor flow field performance. Unlike the outwardly deflected and relatively broad wakes generated by the upper and lower fish bodies, the lateral expansion of the counter-Karman vortex street produced by Fish 2 is strictly suppressed at this spacing. During oscillation, the shed shear layers on both sides of Fish 2 rapidly interact intensely with the adjacent fish’s shear layers in the near-wake region. This close vortex-vortex interference accelerates viscous dissipation of vorticity, causing Fish 2’s wake vortex to decay rapidly in strength during downstream convection at this spacing. Consequently, its wake width is markedly narrower than that of the outer fish. This phenomenon of being tightly “sandwiched” by the flow fields on both sides’ forces Fish 2’s wake to remain centered along the midline, preventing it from undergoing lateral deflection like the outer fish. Consequently, a highly energy-concentrated yet spatially compact, high-dissipation fragmented wake is formed. This results in the middle Fish 2 experiencing the greatest fluid damping at this critical spacing, causing its hydrodynamic performance to plummet to its lowest point.

**Figure 20:**
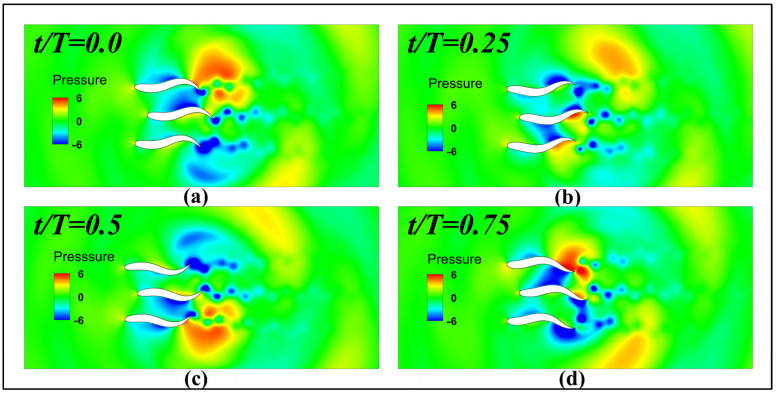
Parallel modal initial spacing 0.4 L periodic pressure contour map during three-fish period instantaneous pressure contour map during period: (**a**) t/T=0.0; (**b**) t/T=0.25; (**c**) t/T=0.5; (**d**) t/T=0.75.

Figure 20 shows the instantaneous pressure contour map for the three fish during one cycle at the initial parallel mode spacing of 0.4 L. At this spacing, the channeling effect between fish bodies reaches its negative peak. Specifically, at t/T = 0.0 and t/T = 0.5, when the tail fins of adjacent fish approach each other, fluid within the extremely narrow gap is violently compressed and cannot escape. This creates a high-pressure zone between the fish, primarily acting on their rear sides and generating significant pressure difference drag. The fish cannot utilize this pressure difference to generate propulsive thrust. At t/T = 0.25 and t/T = 0.75, the gap rapidly expands, creating high negative pressure within the channel. Within a single cycle, the pressure field alternates frequently between both sides of the fish bodies. This causes the middle fish 2 to undergo severe oscillations, not only disrupting the pressure generation mechanism on the fish surface but also forcing the fish to expend additional energy to resist the interference of lateral fluid forces. Consequently, a “high-energy consumption, low-speed” phenomenon occurs.

## 4. Conclusions

This study employs the immersed boundary-lattice Boltzmann numerical simulation method to investigate the autonomous propulsion performance and hydrodynamic mechanisms of multi-fish systems in tandem and parallel formations. By quantifying characteristics such as swimming speed, instantaneous vorticity field, pressure field, and transport cost, the following key conclusions are drawn:

Near-field synergy and far-field divergence effects in tandem swimming: A distinct hydrodynamically advantageous zone exists within tandem formations. When spacing ranges from 1.5 L to 2.0 L, the vortices shed by the leading fish align closely with the head region of the trailing fish, creating positive pressure field synergy. This enables two-, three-, and four-fish systems to achieve the swarm advantage of “high swimming speed with low energy consumption”. As spacing increases to 2.5 L and beyond, flow field synergy significantly diminishes. Middle-row individuals gradually exhibit inefficient swimming, while the lead fish faces severe propulsive resistance and performance loss in the far field. However, for the four-fish long formation, the wake flow field accumulated upstream undergoes secondary reorganization after distant-field convection. This allows the trailing fish at the very end to recapture wake energy, achieving a counter-trend reversal in swimming speed and energy efficiency.

Blockage Mechanism and Critical Instability in Parallel Swimming: Parallel swimming is primarily constrained by the “channel effect” of lateral gap flow. Experiments indicate 0.4 L as the critical threshold for performance degradation within the specific baseline parameters tested (Re = 300, in-phase oscillation). Because this boundary is functionally dependent on transverse wake width and localized vortex interactions, altering phase differences or tail kinematics is expected to shift this instability threshold. At this spacing, intense flow confinement and unsteady disturbances cause propulsion efficiency to plummet for all individuals. While parallel mode generally hinders swimming speed enhancement, appropriate spacing adjustments can maintain formation stability.

Bilateral Constraint Characteristics of Central Individuals in Parallel Modes: Within parallel schools, the fluid environment exhibits significant spatial heterogeneity. Central individuals experience severe lateral spatial compression of their counter-Karman vortices due to the combined effects of bilateral shear layers from adjacent fish, accelerating wake energy dissipation. In contrast, peripheral individuals benefit from unilateral free flow fields and can recover to single-fish swimming levels more rapidly as spacing increases. This finding indicates that when designing large-scale bionic fish formations, spatial layout optimization for internal individuals is crucial to mitigate performance limitations caused by “bilateral blockage”.

Despite the fundamental hydrodynamic insights gained regarding pure wake inter-actions and channel effects, it is imperative to acknowledge the substantial limitations of the current numerical framework. The model is strictly constrained by two-dimensional and single degree of freedom (streamwise only) assumptions. In realistic biological schooling, three-dimensional vortex topological structures (such as interconnected vortex rings and downdraft wakes) play a crucial role in active phase synchronization, spontaneous lateral positioning, and collision avoidance. By suppressing transverse motion, the current model artificially decouples the complex flow-induced posture adjustments naturally observed in fish. Consequently, these specific spatial thresholds should be viewed as fundamental design references for bionic systems, rather than direct representations of unrestricted biological schooling. Rather, they represent the baseline hydrodynamic potential and critical interference mechanisms within rigidly constrained autonomous bionic swarms. Future research must extend to three-dimensional, multi-DOF fluid structure interaction models to accurately capture the fully coupled, spontaneous bio locomotion dynamics of fish schools.

## Figures and Tables

**Table 1:** Comparison results between the mean drag coefficient and the peak lift coefficient for a stationary cylinder at Re = 100.

Project	${\bar{C}}_{D}$	$∆C_{L}$
Present	1.364	0.685
Linnick [34]	1.38	0.674
Kim et al. [35]	1.33	0.640
Liu et al. [36]	1.35	0.678

## Data Availability

The data supporting this study are presented in the article.
